# Genome Sequencing and Comparative Genomics Analysis Revealed Pathogenic Potential in *Penicillium capsulatum* as a Novel Fungal Pathogen Belonging to *Eurotiales*

**DOI:** 10.3389/fmicb.2016.01541

**Published:** 2016-10-05

**Authors:** Ying Yang, Min Chen, Zongwei Li, Abdullah M. S. Al-Hatmi, Sybren de Hoog, Weihua Pan, Qiang Ye, Xiaochen Bo, Zhen Li, Shengqi Wang, Junzhi Wang, Huipeng Chen, Wanqing Liao

**Affiliations:** ^1^Beijing Institute of BiotechnologyBeijing, China; ^2^Beijing Institute of Radiation MedicineBeijing, China; ^3^National Institutes for Food and Drug ControlBeijing, China; ^4^Department of Dermatology, Shanghai Key Laboratory of Molecular Medical Mycology, Shanghai Institute of Medical Mycology, Shanghai Changzheng HospitalShanghai, China; ^5^CBS-KNAW Fungal Biodiversity CentreUtrecht, Netherlands; ^6^Institute of Biodiversity and Ecosystem Dynamics, University of AmsterdamAmsterdam, Netherlands; ^7^Center for Hospital Infection Control, Chinese PLA Institute for Disease Control and PreventionBeijing, China; ^8^Directorate General of Health Services, Ibri Hospital, Ministry of HealthIbri, Oman; ^9^Key Laboratory of the Ministry of Health for Research on Quality and Standardization of Biotech ProductsBeijing, China

**Keywords:** *Penicillium capsulatum*, novel fungal pathogen, genome sequencing, comparative genomics

## Abstract

*Penicillium capsulatum* is a rare *Penicillium* species used in paper manufacturing, but recently it has been reported to cause invasive infection. To research the pathogenicity of the clinical *Penicillium* strain, we sequenced the genomes and transcriptomes of the clinical and environmental strains of *P. capsulatum.* Comparative analyses of these two *P. capsulatum* strains and close related strains belonging to *Eurotiales* were performed. The assembled genome sizes of *P. capsulatum* are approximately 34.4 Mbp in length and encode 11,080 predicted genes. The different isolates of *P. capsulatum* are highly similar, with the exception of several unique genes, INDELs or SNPs in the genes coding for glycosyl hydrolases, amino acid transporters and circumsporozoite protein. A phylogenomic analysis was performed based on the whole genome data of 38 strains belonging to *Eurotiales*. By comparing the whole genome sequences and the virulence-related genes from 20 important related species, including fungal pathogens and non-human pathogens belonging to *Eurotiales*, we found meaningful pathogenicity characteristics between *P. capsulatum* and its closely related species. Our research indicated that *P. capsulatum* may be a neglected opportunistic pathogen. This study is beneficial for mycologists, geneticists and epidemiologists to achieve a deeper understanding of the genetic basis of the role of *P. capsulatum* as a newly reported fungal pathogen.

## Introduction

The term “novel fungal pathogens” has traditionally referred to those non-pathogenic fungi that have been shown to cause human infection, and the number of these pathogens has steadily increased during the last 20 years (Jones et al., 2008). However, the majority of these fungi have been frequently neglected after being reported, although fungal pathogens have recently been recognized as a significant threat to public health (Morens et al., 2004; Garcia-Solache and Casadevall, 2010). Current unprecedented declines in biodiversity may actually increase the risk of fungal infection in mammals, and the potential hazard of novel fungal pathogens cannot be neglected (Keesing et al., 2010; Fisher et al., 2012). Virtually all of these fungal pathogens have been identified using morphological, physiological and multiple locus sequence typing (MLST) analyses (Struelens and Brisse, 2013; Samson et al., 2014). These approaches can identify these fungal pathogens at the species or even the genotype level. However, it is a challenge to investigate the possible causes of variations in the virulence and the evolution, and transmission of novel fungal pathogens using routine identification techniques.

Recently, the application of next-generation sequencing (NGS) technology has allowed us to conduct whole genome scans and to gain insight into the virulence factors and the adaptation of fungal pathogens to human hosts (Pareek et al., 2011; Ferrarini et al., 2013). The second-generation platform Illumina sequencing can provide accurate, high-throughput data with greater sequencing depths, whereas the third generation sequencing platform PacBio RS II can offer increased read lengths and unbiased genome coverage (Ferrarini et al., 2013). Combining the two platforms will produce better sequencing results with increased efficiency. These developments can provide additional insights for obtaining genetic information in novel fungal pathogens to develop new tools for the detection of emergence and the formulation of appropriate responses toward infections, such as infection control. The high levels of variation and unique genomic features of different isolates that are relevant to virulence have been increasingly reported in multiple NGS and comparative studies of human pathogens (Lavezzo et al., 2013). Recent novel phylogenetic findings based on MLST analyses–such as the identification of eurotiomycetous endophytes, which showed close affinities to *Chaetothyriales. Eurotiales*, and the characterization of a new order, *Phaeomoniellales*-still required further phylogenetic proof based on additional genomic information (Chen et al., 2013, 2015). However, similar studies in fungal pathogens have been rare to date (Lavezzo et al., 2013).

Species of *Eurotiales* are common and important to both industry and medicine. Some species, such as *Aspergillus fumigatus*, are well known as human pathogens. *Penicillium* is an important genus belonging phylogenetically to *Eurotiales*. Over 350 species are currently defined in the genus, the majority of which are saprobic and commonly occur in soil. Invasive infections caused by *Penicillium* species are currently rare, and such reports will decrease because *Penicillium marneffei* is now known as *Talaromyces marneffei* after its recent transfer to the genus *Talaromyces*, which belongs to the subgenus *Biverticillium* (Samson et al., 2011). *P. capsulatum*, a rare *Penicillium* species frequently used in the paper-making industry, has never been recognized as a human pathogen (Lyratzopoulos et al., 2002; Houbraken and Samson, 2011). In 2013, we first reported a fungus ball in the left lung of a type 2 diabetic patient caused by *P. capsulatum* (Chen et al., 2013). Routine molecular identification techniques, such as ITS, calmodulin and *RPB2* sequencing (Samson et al., 2014), are insufficient to reveal the hidden genetic basis of the probable cause of novel fungal pathogens such as *P. capsulatum*, and most of these pathogens were reported and neglected.

To further investigate the genomic basis of differences in the pathogenicity of *P. capsulatum* species, we performed genome sequencing and comparative genomics of the clinical (CBS 134186) and environmental (ATCC 48735) strains of *P. capsulatum* using a combination of the second and third generation sequencing platforms. A comparative transcriptome analysis of these two strains was performed using RNA-Seq data. In addition, a new phylogenetic analysis was performed in *Eurotiales* based on the fungal species with available genome data, including *P. capsulatum*. Moreover, we thoroughly researched the potential virulence factors in *P. capsulatum* and its closely related strains, which include both fungal pathogens and non-human pathogens. Our study not only provided additional insights into the pathogenic potential of *P. capsulatum* as an example of novel fungal pathogens belonging to *Eurotiales* but also more accurately explored the phylogenetic placements of *Eurotiales* species.

## Materials and Methods

### Isolates

The clinical strain of *P. capsulatum* (LiaoWQ-2011 = CBS 134186) was isolated from a pulmonary fungus ball in a patient with type 2 diabetes in 2013, and the environmental strain of *P. capsulatum* (ATCC 48735) was isolated from exposed canvas on Gilbert Island in 1945. The representative isolates were included for whole genome sequence analyses in this study, as described in **Supplementary Table S1**.

### Genome DNA and RNA Extraction

The representative strains for *P. capsulatum* (i.e., the clinical strain CBS 134186, and the environmental strain ATCC 48735) were grown on potato dextrose agar (PDA) at 25°C for 14 days. Genomic DNA for whole-genome sequencing was extracted from the pure cultures using the DNeasy Plant Mini kit (QIAGEN Co., Ltd, Hamburg, Germany) according to the instruction manual. RNA for transcriptome sequencing was extracted using the RNeasy Plant Mini kit (QIAGEN Co., Ltd, Hamburg, Germany) following the protocol for the isolation of the total RNA.

### Genome Sequencing

Our strategy for whole-genome sequencing involved a combination of the Illumina MiSeq (Illumina Inc., San Diego, CA, USA) and the Pacific Biosciences RS II (Pacific Biosciences, Menlo Park, CA, USA) sequencing platforms, and the sequence data from the Illumina platform were used to proofread the PacBio assembly sequence. Illumina libraries were prepared using TruSeq DNA sample prep kits (Illumina Inc., San Diego, CA, USA) according to the manufacturer’s instructions. Two paired-end sequencing libraries were constructed. One was a 250 Pair End (PE) sequencing library with an insert sizes of approximately 400 bp, and the other was a 150PE library with insert sizes ranging from 200 bp to 1 kb. Pacific Biosciences RS II sequencing technology was used as the sequencing platform. A 10-kb Single Molecule Real Time (SMRT) bell library was prepared from sheared genomic DNA via a 10-kb template library preparation workflow. SMRT sequencing was conducted on the PacBio RS II sequencing platform using C3 sequencing chemistry and P5 polymerase with 16 SMRT cells.

### Genome Assembly

The Hierarchical Genome Assembly Process (HGAP) was used to assemble these two sequenced genomes. *De novo* assembly of the PacBio read sequences was carried out using continuous long reads (CLR), followed by the HGAP workflow (PacBioDevNet; Pacific Biosciences) as available in SMRT Analysis v2.1. HGAP consists of preassembly, *de novo* assembly with Celera Assembler (CA), and assembly polishing with Quiver. CA software version 7.0 was utilized in the pre-assembly step, and the PacBioRs_PreAssembler with one module and a default minimum subread length of 500 bp, a minimum read quality of 0.80, and a minimum subread length of 7500 bp was used to perform error correction for the raw data generated by the PacBio RS II platform. To polish the assembled sequence from HGAP, the MiSeq read sequences were mapped using BWA v0.7.5a (Li and Durbin, 2009, 2010), and the SNPs and INDELs were called and corrected by SAMtools v0.1.18 (Li et al., 2009) and an in-house script. The genomes of *P. capsulatum* strains CBS 134186 and ATCC 48735 were deposited in Genbank under the accession numbers JPLR00000000 and JPLQ00000000.

### Prediction and Annotation of Gene Structure

InterProScan was used to obtain functional annotations for all predicted genes and to determine motifs and domains. To obtain high confidence gene models for *P. capsulatum*, coding genes were predicted with AUGUSTUS v3.1 (Stanke et al., 2008). All of the predicted gene models were functionally annotated based on their sequence similarity to genes and proteins in the NCBI nucleotide (Nt), non-redundant and UniProt/Swiss-Prot protein databases. The gene models were also annotated based on their protein domains using InterProScan (Jones et al., 2014). All genes were classified according to Gene Ontology (GO), eukaryotic orthologous groups (KOG), and Kyoto Encyclopedia of Genes and Genomes (KEGG) metabolic pathways. Repeat sequences were masked throughout the genome using Repeat Masker v3.2.9 and the RepBase library v16.08 (Jurka et al., 2005). tRNAs were identified using tRNA scan-SE (Lowe and Eddy, 1997; Schattner et al., 2005).

### Orthology and Phylogenetic Analysis

For analysis of orthologous and unique genes, we used the Markov clustering program OrthoMCL v2.09 (Li et al., 2003) to define a gene family as a group of genes that descended from a single gene present in the last common ancestor of the considered species. The peptide sequences were subjected to an all-versus-all BLASTp search with a threshold value of E ≤ 1e–5 and then clustered by MCL with an inflation value of 1.5. A pipeline was used to cluster individual genes into gene families and to analyze orthologous and unique genes. The pipeline included four main steps: (1) data preparation, (2) BlastP searches for all of the protein sequences against a database containing a protein dataset from all species with *E*-values under 1E-5 and a 90% Match Cutoff, (3) clustering by MCL, and (4) extracting gene families.

For the phylogenetic analysis based on all 40 strains, alignments of single-copy orthologs were produced using MAFFT v7.221 (Katoh and Standley, 2013). ProtTestv3.4 (Darriba et al., 2011) was utilized to determine the best-fitting model for the data. A maximum likelihood phylogeny was reconstructed using RAxML v8.1.23 (Stamatakis, 2014). For the phylogenetic analysis based on virulence-related genes, 43 virulence-related genes shared among 22 strains were selected for a similar phylogenetic analysis.

### Secondary Metabolite Genes and Gene Cluster Prediction

The gene clusters related to secondary metabolite biosynthesis were predicted using antiSMASH. All protein sequences were also searched against a local polyketide synthase (PKS)/non-ribosomal peptide synthetase (NRPS) database (a subset of the SwissProt database) using BlastP with a threshold *E*-value ≤ 1E–5 to identify PKS and NRPS. The putative PKS/NRPS protein sequences were further searched against the NCBI Conserved Domain Database (V3.09) to confirm that the three typical domains (PKS, MT, and ACP) were present. The consensus sequences of PKS/NRPS predicted by antiSMASH (Blin et al., 2013) and manually identified proteins were of high confidence.

### Protein Families

Whole genome blast searches were conducted against protein sequences in the pathogen–host interaction database^1^ (*E*-value ≤ 1E-10) (Urban et al., 2015). In the cytochrome P450s analysis, the reference CYP sequences were downloaded from http://drnelson.uthsc.edu/P450seqs.dbs.html. All predicted proteins were then used to search the reference CYP data set using the BLASTP program with a cutoff *E*-value ≤ 1E–10. SignalP (Petersen et al., 2011) was used to predict signal peptide cleavage sites in amino acid sequences, and putative protein sequences were also examined using the TMHMM method (Sonnhammer et al., 1998) for transmembrane domains. The predicted signal peptide sequences without transmembrane domains were identified as exocrine proteins.

### Comparative Genomics Analysis

To identify significantly different genes between the genomes of strains CBS 134186 and ATCC 48735, all of the annotated genes were subjected to all-versus-all BLASTP searches with a threshold *E*-value ≤ 1E–10. Any gene with a coverage rate below 90% was identified as a specific candidate gene. Specific genes were obtained by filtering out genes without a specific annotated function or with any alignments near the gene boundary. Furthermore, comparisons of nucleotide sequences between strains ATCC 48735 and CBS 134186 were carried out for SNP and INDEL detection using the Mauve program with the default values (minimum match length, 20 bp). An in-house script was used to call SNPs and INDELs between strains ATCC 48735 and CBS 134186.

### Synteny Analysis and Visualization

Synteny analysis between ATCC 48735 and CBS 134186 was performed using MUMmer v3.0 (Kurtz et al., 2004) and MAUVE v20150226 (Darling et al., 2010). The two strains of *P. capsulatum* were visualized using the Circos program v0.68 (Krzywinski et al., 2009).

### Transcriptome Sequencing and Assembly

Poly-A mRNA was isolated using oligo-dT-coupled beads from 40 μg total RNA of each sample, followed by shearing. The isolated RNA samples were used for first strand cDNA synthesis with random hexamers and Superscript II reverse transcriptase. After end repair and the addition of a 3’dA overhang, the cDNA was ligated to the Illumina paired-end adapter oligomix, and approximately 200 bp fragments were size selected by gel purification. After 16 cycles of PCR, the libraries were sequenced using Illumina HiSeq2500 and the paired-end sequencing module. The RNA expression analysis was based on the predicted genes of CBS 134186 and ATCC 48735. First, Tophatv2.0.10 (Kim et al., 2013) was used to map the mRNA reads to the genome, and Cufflinksv2.1.1 (Trapnell et al., 2010) was then used to calculate the expected fragments per kilobase of transcript per million mapped reads (FPKM) as the expression values for each transcript. RNA-Seq data of CBS 134186 and ATCC 48735 are available at National Center for Biotechnology Information (NCBI) under SRR accession numbers of SRR 4031065 and SRR 4051963.

## Results

### Genome Sequencing, Assembly, and Assessment

The genomes of both *P. capsulatum* strains were successively sequenced by a combination of the Pacific Biosciences RS II and Illumina MiSeq platforms. A total of 4.9 and 4.4 Gb of raw data were generated for strains CBS 134186 and ATCC 48735, respectively, using the PacBio RSIISMRT platform and a 10-kb library preparation. A total of 3.2 and 3.0 Gb of raw data from strains CBS 134186 and ATCC 48735, respectively, were generated using the Illumina platform. The genome size of *P. capsulatum* was estimated to be approximately 35 Mb. Hence, we sequenced the genomes of the two *P. capsulatum* strains with an average coverage of ∼231 × for strain CBS 134186 and ∼211× for strain ATCC 48735. For strain CBS 134186, the genome size was approximately 34.34 Mb, with a contigN50 size of 3.30 Mb; for strain ATCC 48735, the genome size was approximately 34.37 Mb, with a contigN50 size of 3.19 Mb. The read data from the Illumina platform were used to correct the assembled sequence based on the PacBio platform because the latter normally results in relatively more mistakes (Ferrarini et al., 2013). Fifty SNPs and 23 INDELs were corrected in the genome of strain CBS 134186, whereas 48 SNPs and 29 INDELs were corrected in the genome of strain ATCC 48735. For the corrected SNPs and INDELs, polyN-type errors were the main error type, which accounted for approximately 75 and 73% of the total corrected sides for CBS 134186 and ATCC 48735, respectively. Two different gene footprint coverage methods were applied to validate the coding region coverage of the genome assemblies. The results obtained using the Core Eukaryotic Genes Mapping Approach (CEGMA) showed that 95.56% (237/248) of the core eukaryotic genes that mapped to the assembly genome of CBS 134186 were identified and that 97.18% (241/248) of the eukaryotic genes that mapped to the genome of ATCC 48735 were identified (**Table 1**).

**Table 1:** Genome characteristics of *Penicillium capsulatum* strains CBS 134186 and ATCC 48735.

Nuclear genome	CBS 134186	ATCC 48735
Size (Mb)	34.341	34.374
Coverage (fold)	231x	211x
Contigs	62	65
N50 length (bp)	3304607	3188669
N50 contig	5	5
G+C content (%)	49.058%	49.074%
Number of protein-coding genes (> 30aa)	11,080	11076
Average gene length (bp)	1807.8	1808.7
GC content of protein-coding genes (%)	52%	52%
Mean number of exons/gene	3.3	3.3
Average length of exons (bp)	482.3	482.5
Mean number of introns/gene	2.3	2.3
Average length of introns (bp)	102.8	103.3
tRNA number	185	174
Repetitive DNA (%)	4.19 %	4.22%
Genbank accession number	JPLR00000000	JPLQ00000000


### Functional Annotation and Genome Features

For the genome of strain CBS 134186, a total of 11,080 gene models were identified based on Gene Ontology (GO) annotations, with an average coding sequence length of 1807.8 bp. For the ATCC 48735 genome, 11,076 gene models were obtained, with a total length of 34.37 Mb and an average coding sequence length of 1808.7 bp (**Table 1**; **Supplementary Table S2**). The average GC content in both strains of *P. capsulatum* was 49.06%. Approximately 1.41 Mb of repeated regions were found in the genome of the clinical strain CBS 134186, which accounted for 4.19% of its genome size, whereas 1.42 Mb of repeated regions were found in the genome of the ATCC 48735 strain. For both strains, interspersed repeats were the predominant type of repeat region, accounting for 84.9% and 85.1% of the repeat regions in the CBS 134186 and ATCC 48735 genomes, respectively. In addition, tRNAScan-SE predicted 185 and 174 tRNAs in the genomes of CBS 134186 and ATCC 48735, respectively.

### Phylogenetic Analysis

A phylogenetic analysis based on the whole genome sequence was conducted to determine the relationship of *P. capsulatum* with other important species belonging to *Eurotiales.* The included species were pre-divided into four categories: *Eurotiales* species known to be medically important fungal pathogens; opportunistic fungal pathogens; species newly reported to cause invasive infection; and species unreported as human pathogens. Except for the sequencing data of *P. capsulatum*, other publicly available and complete genomic data of *Eurotiales* were downloaded from NCBI, including those data for thirteen *Penicillium* strains, fourteen *Aspergillus* strains, five *Talaromyces* strains, two *Neosartorya* strains, one *Rasamsonia* strain and one *Byssochlamys* strain. Two *zygomycete* pathogens, *Rhizopus oryzae* and *Mucor circinelloides*, were selected as taxonomic out-groups (**Supplementary Table S1**).

**FIGURE 1:**
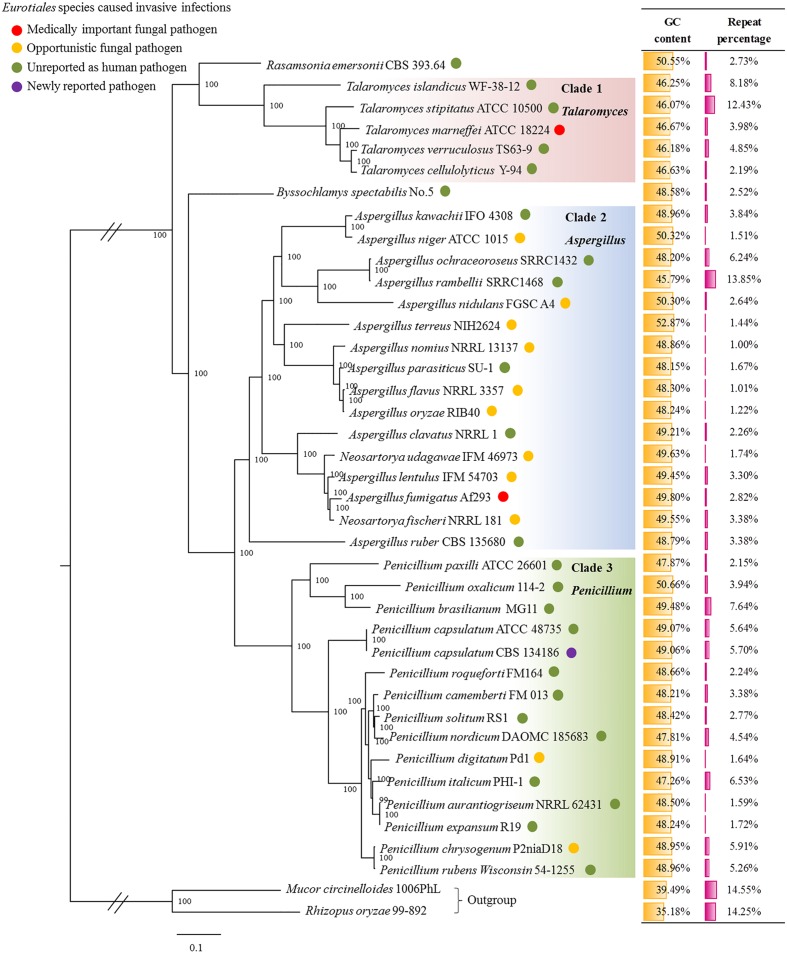
**Phylogenetic analysis of *Eurotiales* based on all of the single-copy orthologues among 40 whole genome sequences.** Maximum Likelihood tree was inferred by RAxML based on single-copy orthologs with best model JTT+I+G+F identified using ProtTest. The tree was rooted with *Mucor circinelloides* and *Rhizopus oryzae*. Bootstrap percentages of the maximum likelihood analysis were presented at the nodes.

The genomes ranged in size from 26.0 to 35.9 Mb within *Penicillium*, from 24.2 to 39.5 Mb within *Aspergillus*, from 28.6 to 37.6 Mb within *Talaromyces* and from 31.7 to 32.2 Mb within *Neosartorya.* The genome sizes of *Rasamsonia emersonii* and *Byssochlamys spectabilis*, which belong phylogenetically to *Eurotiales*, were 28.2 and 29.8 Mb, respectively. Within *Eurotiales. A. rambellii*, and *T. stipitatus* carried significantly higher percentages of repetitive elements (13.85 and 12.43%, respectively). The number of repetitive sequences of other strains in *Eurotiales* was relatively low; only 1.01-8.18% of each genome was classified as repetitive. In contrast to the low GC content in the out-group, the genomic GC content of *Eurotiales* ranged from 46 to 53% with a balanced ratio (**Figure 1**).

Subsequently, all of the single-copy orthologs were selected for the phylogenetic analysis. *Eurotiales* were divided roughly into three groups, in which *Penicillium* had a closer phylogenetic relationship to *Aspergillu* than to *Talaromyces.* Fifteen *Penicillium* strains were grouped together in one clade, suggesting their origin from a single progenitor species (**Figure 1**). The *P. capsulatum* strains were confidently placed in a basal position to *P. chrysogenum* and other species belonging to the subgenus *Penicillium*. The clinical strain of *P. capsulatum* showed a very close phylogenetic relationship with the environmental strain, forming a subclade within the *Penicillum* clade. *Talaromyces marneffei*, which was previously known as *P. marneffei*, was grouped in the genus *Talaromyces* based on its genomic data in this study. In addition, *Neosartorya* was classified with *Aspergillu* into one clade according to their genomic characteristics. These results offered us a new perspective based on whole genome data for future phylogenetic classifications.

### Analysis of Virulence-Related Genes in *P. capsulatum* and Related Species

To further explore the pathogenic factors of *P. capsulatum* and its closely related strains, we performed a phylogenetic analysis based on 106 virulence-related genes identified from medically important fungal pathogens, such as *A. fumigatus. T. marneffei et al.* as references for studying the novel fungal pathogens (**Supplementary Table S3**). Except for two *P. capsulatum* strains, 18 species were selected according to their medical or economic importance and their phylogenetic position, with the intention to obtain a focused representation of *Eurotiales*. The following *Eurotiales* species were included: species known to frequently cause invasive infections as medically important fungal pathogens, including *A. fumigates* and *T. marneffei*; species reported as opportunistic fungal pathogens, including *A. niger. A. nidulans. A. terreus. A. flavus. A. oryzae. P. chrysogenum. P. oxalicum. P. digitatum*, and *N. fischeri*; and species unreported as human pathogens, including *T. stipitatus. A. clavatus. P. camemberti. P. paxilli. P. aurantiogriseum. P. expansum*, and *P. rubens Wisconsin. Rhizopus oryzae* and *Mucor circinelloides* were included as taxonomic out-groups.

**FIGURE 2:**
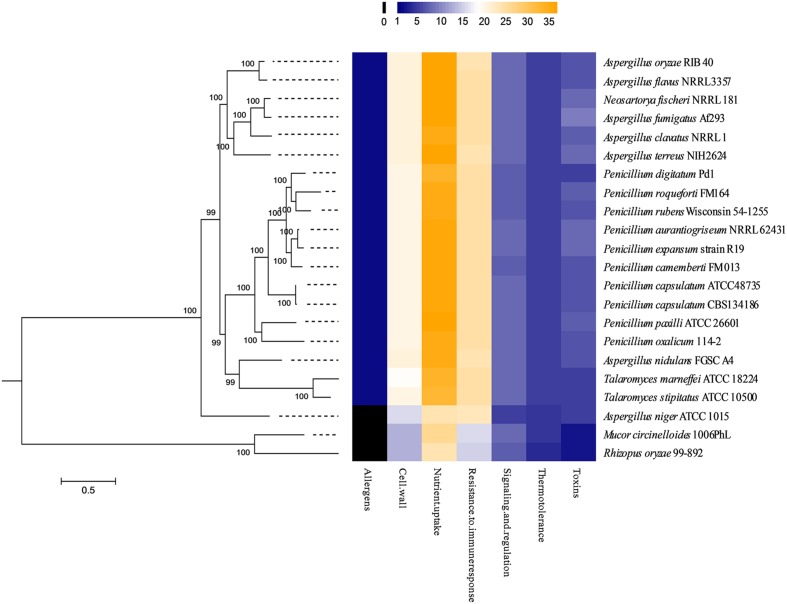
**Phylogenetic analysis based on 43 virulence-related genes shared among 22 related species.** The heat map on the right represented the number of virulence-related genes in each strain.

The presence or absence of virulence-related genes was searched in the 22 studied genomes. We found that a total of 43 virulence-related genes were shared the studied genomes. These genes could be divided into the following six categories: toxins, thermo-tolerance, signaling and regulation, resistance to immune response, cell wall, allergens and nutrient uptake (**Supplementary Table S4**). Generally, the medically important fungal pathogens have more virulence-related genes than non-human pathogens. In this study, the clinical and environmental isolates of *P. capsulatum* were found to be very similar in terms of their virulence-related genes. Surprisingly, we found in this phylogenic analysis that *P. paxilli*, which is used as a model to study the biochemistry of indol-diterepene biosysnthesis and is seldom reported to be infectious to humans, had a high proportion of virulence-related genes and very close relationship to *P. capsulatum* (**Figure 2**). Thus, we selected *P. paxilli* and two well-known human pathogens, *A. fumigates* and *T. marneffei*, for a more detailed comparison with *P. capsulatum.*

### Comparison and Analysis Among the Four Related Strains

**FIGURE 3:**
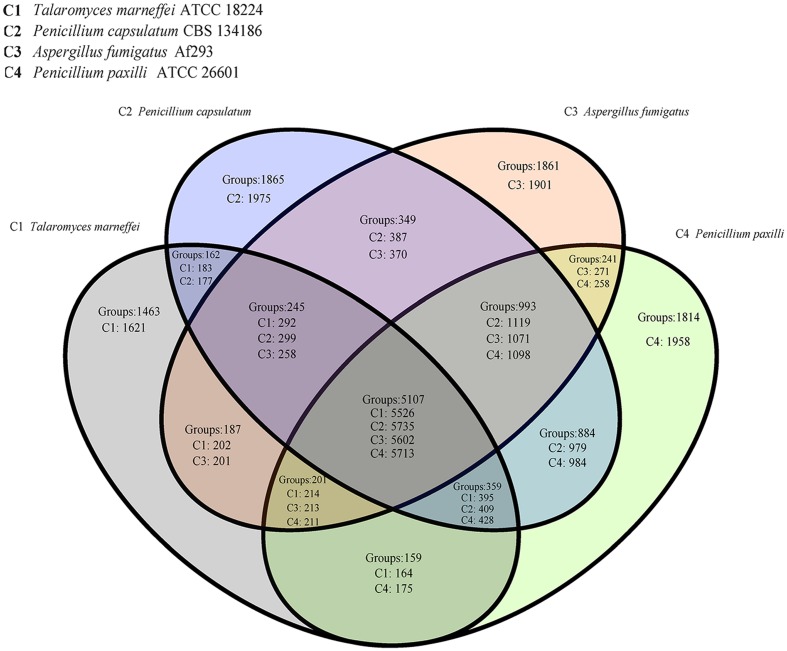
**Venn diagram showing shared orthologous groups among genomes of *Talaromyces marneffei. P. capsulatum. Aspergillus fumigatus. P. paxilli*.** The numbers marked behind “Groups” in each region represent for orthologous groups, and the numbers following C1∼C4 means the counts of genes in the strains.

Using a Venn diagram of four strains, we compared the *P. capsulatum* genome with a non-human pathogen, *P. paxilli*, which had a close phylogenetic relationship to *P. capsulatum*, as well as *A. fumigates* and *T. marneffei*, two major human pathogens. By analyzing the homologous genes among the four genomes and enriching the virulence genes among these groups, as expected, the majority of homologous gene groups were in the groups of C1&C2&C3&C4. In this shared group among all four strains, most genes were identified as belonging to the cytochrome P450 (CYP) family and the major facilitator superfamily (MFS), which generally play important roles in the biosynthesis and transportation of metabolites. The second largest group of genes shared by these strains was group C2&C3&C4, indicating that the relationships among *P. capsulatum. P. paxilli*, and *A. fumigate* were closer than *T. marneffei*. Compared with the other three strains, the unique paralog gene groups of *A. fumigate* in group C3 consisted of typical virulence-related genes, such as genes involved in gliotoxin synthesis (*GliZ. Glip*) and fumitremorgin synthesis (*ftmE-H*). Like group C3, group C2 comprised unique paralog gene groups found in *P. capsulatum* CBS 134186. This group had the most unique paralog gene groups, containing 1865 gene groups (1975 predicted genes in total). Some of these genes may increase the pathogenic capacity of these four strains (**Figure 3**, **Supplementary Table S5**).

By further investigating the virulence genes, we found that only 7 out of 15 groups contained virulence-related genes (**Supplementary Table S6**). The majority of the virulence genes (*n* = 75) were in the group C1&C2&C3&C4, which had virulence genes among all the four strains. There were 13 virulence factors in group C2&C3&C4, which contained the second greatest number of virulence genes. Interestingly, group C3&C4 contained four virulence genes, which was greater than the number of virulence genes in group C1&C3 and group C2&C3. The enrichment of virulence-related genes in *P. paxilli* indicated the need for paying increased attention to its potential pathogenicity. However, it is possible that having these virulence-related genes is not sufficient to cause disease. Thus, the underlying difference between fungal pathogens and non-pathogens needs further study.

### Comparative Genomic Analysis of *P. capsulatum* Strains CBS 134186 and ATCC 48735

**FIGURE 4:**
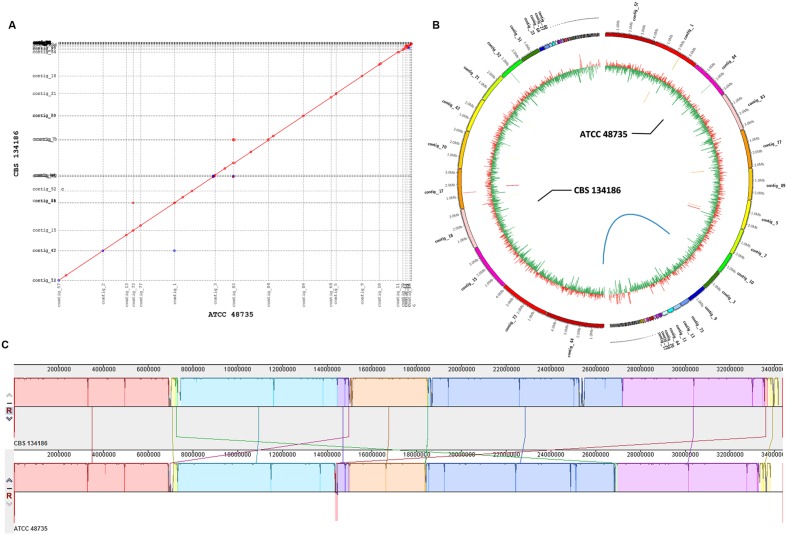
**Genome comparison of strains CBS 134186 and ATCC 48735.** **(A)** Collinearity plot. The forward matches were displayed in red, while the reverse matches were displayed in blue. **(B)** Difference between two strains. The left part of the circle indicates CBS 134186 and the right part indicates ATCC 48735. Rings from the outermost to the center: (1) Scale marks of the genome. (2) SNPs. Bases of A, T, G, and C were represented in red, blue, green, and yellow. (3) GC content. The ratio of GC sites per 10 kb were plotted in red (> = 50%) and green (<50%). (4) Special genes. Blastp similarity ranged from 100% (blue) to 0% (red). Genes with lower sequence similarity tended to hotter orange and red tones. (5) INDELs in blue links. **(C)** Mauve plot of two strains. Collinear contigs were sorted in proper order.

The genome of strain CBS 134186 was 0.033 Mb smaller than the genome of ATCC 48735 (**Table 1**). We speculated that the gaps between the large scaffolds in the CBS 134186 genome (*n* = 104) and the ATCC 48735 genome (*n* = 101) may be responsible for the different genome sizes observed between these two strains. Pair-wise genome alignments showed that the two *P. capsulatum* strains were highly syntenic and shared, on average, 99.98% sequence similarity (**Figures 4A,C**). These results suggested that the pathogenic potential of the clinical strain CBS 134186 and the environmental strain ATCC 48735 may be extremely similar or that the specific genes, SNPs or INDELs present in the CBS 134186 genome may play a key role in causing invasive infection. Among these probable mutations, a total of seven unique genes, SNPs or INDELs in the CBS134186 genome may be involved in the cause of make itself to be a novel fungal pathogen (**Table 2**, Supplementary Tables S7-S9; **Figure 4B**). Cytochrome P450, secondary metabolism, and exocrine proteins analyses were also performed on these seven genes. One of the unique genes (g512) was predicted as an exocrine protein gene; there were no positive results in the secondary metabolite and cytochrome P450 analyses. The unique genes coding for vegetative incompatibility protein and glycosyl hydrolases (g512 in contig27), the SNP in the gene coding for circumsporozoite protein (g399 in contig17), and the INDEL in the gene containing the F-actin capping protein beta subunit (g8270 in contig44) in the CBS 134186 genome may be related to its adaptation to stress conditions; a similar phenomenon has been observed in the interaction between *A. fumigates* and humans (Abad et al., 2010).

**Table 2:** The unique characteristics with pathogenic potential in strain CBS 134186 genome by compared with the environmental strains.

Gene ID	Mutation location	Function	Exocrine protein gene	PHI database	Type	Secondary metabolite	Cytochrome P450s
g97	contig_17:376853-381151	Vegetative incompatibility protein HET-E-1	No	PHI: 211	gene	No	No
g10464	contig_70:1865570-1869484	N amino acid transport system protein	No	PHI: 511	gene	No	No
g512	contig_17:1693336-1696043	1,3-beta-glucanosyltransferase gel3	Yes	PHI: 522	gene	No	No
g399	contig_17:1308904	Circumsporozoite protein	No	—	SNP	No	No
g1265	contig_52:720079	Zn(2)-C6 fungal-type DNA-binding domain	No	PHI: 1163	SNP	No	No
g1265	contig_52:720280	Zn(2)-C6 fungal-type DNA-binding domain	No	PHI: 1163	SNP	No	No
g8270	contig_44:191141	F-actin-capping protein subunit beta	No	PHI: 2568	INDEL	No	No


### Comparative Transcriptomic Analysis of Strains CBS 134186 and ATCC 48735

To more accurately annotate the *P. capsulatum* genome, we performed deep transcriptome sequencing of both *P. capsulatum* strains (CBS 134186 and ATCC 48735), which generated 4.0 and 2.9 Gb of RNA sequencing data, respectively. Compared with ATCC 48735, CBS 134186 contained 481 differentially expressed genes and two up-regulated virulence-related genes, which were observed under *in vitro* culture conditions by three replicates to give a general estimate. These virulence-related genes were beta-(1,3) glucanosyltransferase (GEL-3) and mannose-6 phosphate isomerase (MPI), which were up-regulated fivefold and threefold, respectively. Moreover, these two genes are involved in cell wall composition and maintenance, and the high expression of these genes may increase the pathogenicity of the clinical strain CBS 134186.

### Morphological Characteristics of Strains CBS 134186 and ATCC 48735

**FIGURE 5:**
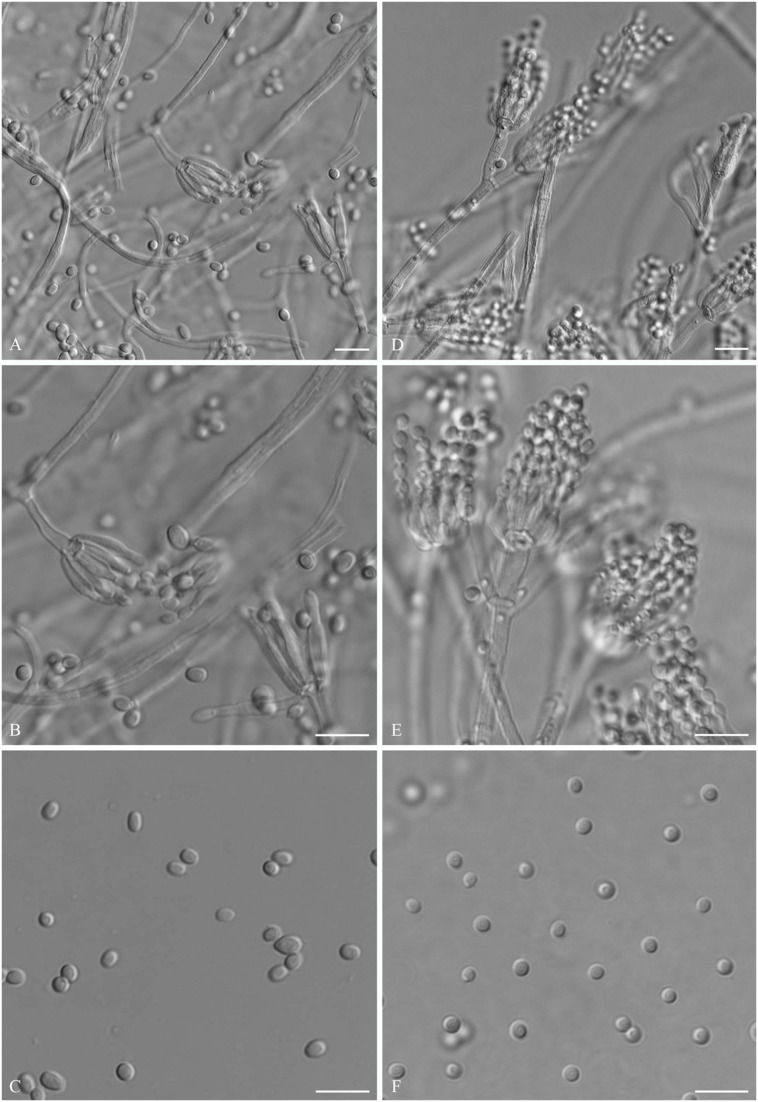
**Comparison of the differences of morphological characteristics between two *P. capsulatum* strains CBS 134186 and ATCC 48735, which were cultured in 25°C on PDA medium.** (magnification ×63 for **(A,D)**, magnification ×100 for **(B,C,E,F)**, scale bars = 10 μm).

We comparatively analyzed the morphological characteristics between strain CBS 134186 and strain ATCC 48735. Both *P. capsulatum* strains had smooth walled conidia that were ellipsoid to slightly cylindrical in shape (**Figure 5**). There were no significantly different morphological characteristics between the two strains, which was in accordance with their highly similar genomes.

## Discussion

In the present study, the genomes of two *P. capsulatum* strains were successively sequenced using the Pacific Biosciences RS II and Illumina MiSeq platforms in combination. There are only 5 contigs length beyond N50 in CBS 134186 and ATCC 48735, which demonstrated the high integrity of the genome assembly. Our assembly size of the genome is similar to reported *Penicillium* genome (van den Berg et al., 2008). Furthermore, the combined application of these second and third generation sequencing technologies shows good potential for the development of genome sequence assemblies without reference to genomic data, thus resulting in fewer unresolved gaps or ambiguities and a smaller number of contigs.

To explore the pathogenic potential of novel pathogens in *Eurotiales*, such as *P. capsulatum*, we referred to studies of representative fungal pathogens, such as *A. fumigatus, T. marneffei*, and others. As successful fungal pathogens, numerous genes from these species have been systematically researched and verified to be involved in virulence (Pitt, 1994; Abad et al., 2010; Chakrabarti and Slavin, 2011; Sheppard and Filler, 2015). A total of 106 genes were selected after we systematically reviewed the virulence-related genes of these important fungal pathogens (**Supplementary Table S3**). Then, we conducted a further analysis based on the homologous virulence-related genes between *P. capsulatum* and other related *Eurotiales* species. These genes can be grouped into seven main functional aspects: thermo-tolerance, cell wall composition and maintenance, resistance to the immune response, toxins, nutrient uptake during invasive growth, signaling, metabolic regulation and response to stress conditions, and allergens. Moreover, these virulence-related genes are considered to result from a combination of biological characteristics of the fungus and the immune status of the patient. Interestingly, a considerable number of virulence-related genes are shared between novel fungal pathogens, such as *P. capsulatum*, and non-pathogenic fungi, such as *P. paxilli*. This suggests the pathogenic potential of these *Penicillium* species, even if some of these species have not yet been reported as human pathogens. Certainly, the bioinformatics analysis has its inherent limitation to predict fungal pathogenicity, further verification and analysis works are needed. Here, we just make a discussion on the pathogenicity evaluation based on the whole genome sequencing data.

A total of 101 possible virulence-related genes were found in the *P. capsulatum* genome (**Supplementary Table S10**). Of these, we selected some important probable virulence-related genes for further analysis with regard to seven aspects. First, thermo-tolerance is considered to be an essential ability for a fungal pathogen to successfully infect humans. A total of five genes have been found to be essential for thermo-tolerance, including the *thtA. afpmt1. kre2*/*afpmt1. cgrA*, and *hsp1*/*asp f12* genes. All of these genes were identified in both strains CBS 134186 and ATCC 48735. Among these genes, *kre2*/*afpmt1* and *cgrA* genes are required for growth at 48C and 37°C, respectively; notably, the protein encoded by *kre2*/*afpmt1* has also been considered as a new antifungal target (Bhabhra et al., 2004; Wagener et al., 2008). Notably, contig_42g3968 and contig_21g7395 in CBS 134186 were regarded as the *kre2*/*afpmt1* and *cgrA* genes by functional annotation and verification using the pathogen-host interactions database. Similar results were obtained in the genome of ATCC 48735, it showed potential thermo-tolerance, which may be crucial for a novel fungal pathogen. A previous study by our group also showed that CBS 134186 grows well in MEA, CYA, and YES cultures at 37°C, and grows faster at 30 and 37°C than at 25°C (Chen et al., 2013). Second, due to its structural integrity and physical protection, the fungal cell-wall is the mainline of defense against the human inner environment and is frequently attacked as a target of the immune system during infection. A total of 20 genes, including *fks1. gel1*, and *ecm33*, have been confirmed as necessary for the composition and maintenance of the cell wall (Mouyna et al., 2000a,b, 2004; Chabane et al., 2006; Maubon et al., 2006; Romano et al., 2006; Wagener et al., 2008; Gastebois et al., 2009). These genes were also found in the studied *Penicillium* species, including *P. capsulatum*, suggesting that the composition and maintenance of the cell wall play important roles during infection. Third, a successful fungal pathogen can evade or resist the immune response and can also weaken the host immune response. A total of 25 genes, including *pksP*/*alb1* and *cat2*/*katG*, have been found to be associated with resistance to the human immune response in previous studies (Calera et al., 1997; Langfelder et al., 1998; Brakhage and Liebmann, 2005; Tsitsigiannis et al., 2005; Reeves et al., 2006; Abad et al., 2010; Al-Bader et al., 2010). Interestingly, all 25 of these genes have been found to be similar to genes in the *P. capsulatum*. For example, the *cat2*/*katG* gene in the *A. fumigates* genome encodes the hyphal catalases, and the deletion of this gene resulted in increased susceptibility of conidia to H_2_O_2_ in *vitro* (Abad et al., 2010). Our results implied that the profile of resistance to the immune response in *P. capsulatum* may be similar to that of *A. fumigates* during infection, which has a combination of characteristics that helps the fungus to evade or resist to immune response (Abad et al., 2010). Fourth, toxins produced by fungi not only protect the fungi from predators and competitors in their ecological niche (Abad et al., 2010; Steinbach et al., 2006) but may also contribute to the pathogenesis of fungal pathogens, such as *A. fumigates*, because they can directly attack the host. No fewer than nine genes in *A. fumigates* have been shown to attack humans, and most of these genes are associated with secondary metabolites (Abad et al., 2010). Six of these nine genes have homologues in *P. capsulatum* that are also related to secondary metabolites. Hence, *P. capsulatum* may produce similar toxins that can attack humans. Fifth, the uptake of nutrients in the human internal environment is an essential for the success of a fungal pathogen (Abad et al., 2010). Currently, no fewer than 37 genes have been associated with the uptake of nutrients in the human internal environment, including through normal nutrient uptake systems and other activated systems (D’Enfert et al., 1996; Brown et al., 2000; Hissen et al., 2005; Moreno et al., 2007; Schrettl et al., 2007; Al-Bader et al., 2010; Amich et al., 2010; Li et al., 2016). Thirty-six of these 37 homologous genes have been found in *P. capsulatum*. Our results strongly suggested that *P. capsulatum* may have a considerable capacity for nutrient uptake in the human internal environment. Sixth, medically important fungal pathogens can regulate their cellular physiology to adapt to the various, dynamic, and changing conditions relative to the normal environmental niche during infection. These conditions include increased osmolarity, heatshock and nutrient limitation, and adaptation can occur through several defined signaling regulatory mechanisms, such as the mitogen-activated protein kinase (MAPK) pathways, Rasproteins, calcium signaling, and others. To date, no fewer than eight genes have been confirmed to be related to the regulation of signaling during adaptation to the environment in association with infection in the previous reports (May et al., 2005; Abad et al., 2010). Interestingly, all of these eight genes have been found to be similar to genes present in *P. capsulatum* strains. Among these genes, *tcsB. Sln1. calA*, and *cnaA* have been shown to be related to stress responses through, for instance, the signaling regulation of adaptation by Ras-proteins or MAPK pathways; notably, the deletion of genes involved in this pathway in fungal pathogens results in hypo-virulence (Pott et al., 2000; Clemons et al., 2002; Steinbach et al., 2006, 2007). Seventh, most fungal pathogens can produce various allergenic molecules, but they can also increase the risk associated with aspergillosis because they can redirect the immune response against the fungus by activating *Th2* lymphocytes (Reichard et al., 1990; Kolattukudy et al., 1993; Tang et al., 1993; Jaton-Ogay et al., 1994). We searched the homologous genes of *P. capsulatum*, and not surprisingly, similar genes, such as *asp f*13, were also detected in *P. capsulatum* strains. Again, this result suggested the pathogenic potential of *P. capsulatum* as a novel fungal pathogen.

The above virulence-related genes were observed in equal numbers in the CBS 134186 and ATCC 48735 genomes, which not only suggested the pathogenic potential of *P. capsulatum* as a novel fungal pathogen but also implied that the difference in the pathogenic potential of novel fungal pathogens between the clinical and environmental isolates may be very small compared with our expectations. We suppose the absence of virulence-related genes in *P. capsulatum* may play a key role in the divergence between novel fungal pathogens and medically important fungal pathogens.

To better study the differences between the clinical and environmental isolates of *P. capsulatum*, we performed a comparative genomic analysis of strains CBS 134186 and ATCC 48735. The results showed that the pathogenic potential of the clinical strain CBS 134186 and environmental strain ATCC 48735 may be extremely similar and that those specific genes, SNPs or INDELs present in the CBS 134186 genome may play a key role in causing invasive infection. Among these probable mutations, a total of 7 unique genes, SNPs or INDELs in the CBS 134186 genome were as likely candidates for conferring the pathogenic status needed to make this strain a novel fungal pathogen. The unique genes coding for vegetative incompatibility protein and glycosyl hydrolases (g512 in contig27), the SNP in the gene coding for circumsporozoite protein (g399 in contig17), and the INDEL in the gene containing F-actin capping protein beta subunit (g8270 in contig44) in the CBS 134186 genome may be related to its adaptation to stress conditions, as observed in the interaction between *A. fumigates* and humans. Moreover, a transcriptomic analysis showed that the g512 and g9805 genes in CBS 134186 were expressed at significantly higher levels than their respective homologs in ATCC 48735 (g3527 and g10066). Both of these genes have been confirmed to be required for the composition and maintenance of the cell wall in *A. fumigates* (Mouyna et al., 2000a,b, 2004, 2005; Chabane et al., 2006; Maubon et al., 2006; Romano et al., 2006; Gastebois et al., 2009). We also speculate these two genes might help the clinical strain to adapt to the human internal environment, as observed in the interaction between *A. fumigates* and humans (Wagener et al., 2008; Abad et al., 2010). These results are likely applicable beyond the present study of *P. capsulatum* and should be considered for other novel fungal pathogens.

## Conclusion

We first report the *P. capsulatum* genome by using a combined NGS strategy. A phylogenetic analysis based on whole genome data may offer us comprehensive information of each strain. The analysis of virulence-related genes in *P. capsulatum* and related species revealed that these strains have significant pathogenic potential. In addition, the comparative genomic analysis of the *P. capsulatum* strains showed high similarity between the clinical strain CBS 134186 and the environmental strain ATCC 48735, except for several unique genes, INDELs or SNPs in genes such as that which codes for circumsporozoite protein; these differences may increase the infectious capacity of this clinical strain. Remarkably, a number of virulence-related genes (such as *gel1-3. mirB*, and *afpmt1* in *A. fumigatus*) in CBS 134186, which have also been identified in the *P. capsulatum* genome, may be closely related to the pathogenicity of *P. capsulatum* and the phylogenetically related *Penicillium* species. Moreover, considering the host of CBS 134186 is a type 2 diabetes patient, the inner-host environment, glucose metabolism and host immune status may be different, which could also increase the possibility of *P. capsulatum* infection. We believe that this study deepens our understanding of the genomic features of *P. capsulatum* and reveals a considerable level of pathogenic potential in *P. capsulatum* as an example of a novel but easily neglected *Penicillium* pathogen. Clinicians, mycologists and epidemiologists should be aware of novel fungal pathogens such as *P. capsulatum*.

## Author Contributions

WL, JW, HC, and SW conceived the project. YY, MC, and ZL prepared the strain samples and did the sequencing test. ZL, XB conducted the bioinformatics analysis. MC, YY, and ZL prepared the manuscript. AA, GdH, WP, QY, and YL participated in discussions and provided suggestions. All authors read and approved the final manuscript.

## Conflict of Interest Statement

The authors declare that the research was conducted in the absence of any commercial or financial relationships that could be construed as a potential conflict of interest.

## Footnotes

1 http://www.phi-base.org/
